# Targeted Deletion of Glycoprotein B Gene by CRISPR/Cas9 Nuclease Inhibits *Gallid herpesvirus* Type 3 in Dually Infected Marek’s Disease Virus-Transformed Lymphoblastoid Cell Line MSB-1

**DOI:** 10.1128/jvi.02027-21

**Published:** 2022-03-23

**Authors:** Yaoyao Zhang, Weicheng Li, Na Tang, Katy Moffat, Venugopal Nair, Yongxiu Yao

**Affiliations:** a The Pirbright Institute, Pirbright, Guildford, Surrey, United Kingdom; b Shandong Binzhou Animal Science and Veterinary Medicine Academy & UK-China Centre of Excellence for Research on Avian Diseases, Binzhou, Shandong, PR China; c The Jenner Institute Laboratories, University of Oxford, Oxford, United Kingdom; d Department of Zoology, University of Oxford, Oxford, United Kingdom; Lerner Research Institute, Cleveland Clinic

**Keywords:** CRISPR/Cas9 editing, MDV-transformed cell line, Marek’s disease virus, gB

## Abstract

Marek’s disease virus (MDV) is a member of the genus Mardivirus in the subfamily *Alphaherpesvirinae*. There are three different serotypes of MDV designated as MDV-1 (*Gallid herpesvirus* type 2), MDV-2 (*Gallid herpesvirus* type 3), and MDV-3 (Meleagrid herpesvirus 1, herpesvirus of turkeys, HVT). MDV-1 is the only serotype that induces Marek’s disease (MD), a lymphoproliferative disorder resulting in aggressive T-cell lymphomas and paralytic symptoms. In the lymphomas and lymphoblastoid cell lines (LCL) derived from them, MDV establishes latent infection with limited viral gene expression. The latent viral genome in LCL can be activated by co-cultivation with chicken embryo fibroblast (CEF) monolayers. MSB-1, one of the first MDV-transformed LCL established from the splenic lymphoma, is distinct in harboring both the oncogenic MDV-1 and non-oncogenic MDV-2 viruses. Following the successful application of CRISPR/Cas9 editing approach for precise knockdown of the MDV-1 genes in LCL, we describe here the targeted deletion of MDV-2 glycoprotein B (gB) in MSB-1 cells. Due to the essential nature of gB for infectivity, the production of MDV-2 plaques on CEF was completely abolished in the MDV-2-gB-deleted MSB-1 cells. Our study has demonstrated that the CRISPR/Cas9 system can be used for targeted inactivation of the co-infecting MDV-2 without affecting the MDV-1 in the MSB-1 cell line. Successful inactivation of MDV-2 demonstrated here also points toward the possibility of using targeted gene editing as an antiviral strategy against pathogenic MDV-1 and other viruses infecting chickens.

**IMPORTANCE** Marek’s disease (MD) is a lymphoproliferative disease of chickens characterized by rapid-onset lymphomas in multiple organs and by infiltration into peripheral nerves, causing paralysis. Lymphoblastoid cell lines (LCL) derived from MD lymphomas have served as valuable resources to improve understanding of distinct aspects of virus-host interactions in transformed cells including transformation, latency, and reactivation. MDV-transformed LCL MSB-1, derived from spleen lymphoma induced by the BC-1 strain of MDV, has a unique feature of harboring an additional non-pathogenic MDV-2 strain HPRS-24. By targeted deletion of essential gene glycoprotein B from the MDV-2 genome within the MSB-1 cells, we demonstrated the total inhibition of MDV-2 virus replication on co-cultivated CEF, with no effect on MDV-1 replication. The identified viral genes critical for reactivation/inhibition of viruses will be useful as targets for development of *de novo* disease resistance in chickens to avian pathogens.

## INTRODUCTION

Marek’s disease virus (MDV-1, *Gallid herpesvirus* 2, GaHV-2), the causative agent of Marek’s disease (MD), is an oncogenic herpesvirus (1) inducing complex clinical syndromes in chickens including immune suppression, paralysis associated with neuronal lymphocytic infiltration, and the rapid-onset CD4^+^ T-cell lymphomas (2–4). Genus Mardivirus, where MDV-1 belongs, also include antigenically related non-pathogenic MDV-2 (GaHV-3) and herpesvirus of turkey (HVT, MeHV-1), isolated from chickens and turkeys, respectively. MD has been controlled for 5 decades by the widespread use of live attenuated vaccines (5, 6) that include the naturally attenuated MDV-1 strain Rispens (CVI988), MDV-2 strain SB-1, and HVT strain FC126. One of the major challenges facing the vaccination strategy is the evolution of viruses toward greater virulence, forcing the need to introduce newer vaccines or alternative intervention strategies to keep up with rapidly evolving viruses.

The CRISPR (clustered regularly interspaced short palindromic repeat)/Cas9 system has become a powerful gene editing tool with many applications in biology, including manipulation of genomes of several large DNA viruses (7, 8) such as herpes simplex (9), pseudorabies (10–13), vaccinia (14), Epstein-Barr virus (EBV) (15), guinea pig cytomegalovirus (16), duck enteritis virus (17), HVT (18, 19), and MDV-1 (20–22). CRISPR/Cas9 editing has also been used in the targeted editing of the latent genomes of herpesviruses. For example, it has been shown that EBV can be efficiently cleared from EBV-transformed human cell lines by targeting of essential genetic elements of EBV (23). Reduction of Kaposi’s Sarcoma-associated herpesvirus (KSHV) latency has also been demonstrated by editing the latency-associated nuclear antigen gene (24). In addition, CRISPR/Cas9 editing has also been used to target the integrated HIV-1 proviral genomes to reduce HIV-1 infection and clear the provirus, as well as to induce transcriptional activation of latent virus in latent viral reservoirs for elimination (25–28).

As clonal populations of transformed tumor cells with latent MDV genome and limited viral gene expression (29–31), lymphoblastoid cell lines (LCLs) derived from MD lymphomas have served as valuable resources to study virus-host molecular interactions in transformed cells. However, detailed investigations into the functional role of different viral and host determinants in these cells have been difficult due to the lack of tools for *in situ* manipulation of viral/host genomes in MDV-transformed cell lines. Our recent success in efficient CRISPR/Cas9 editing of the MDV genome in LCLs such as MDCC-HP8 and MSB-1 has demonstrated the potential for targeted editing of the host and viral genes to dissect the regulatory pathways associated with latency, transformation, reactivation and lytic switch (20, 21).

The MDV-transformed LCL MSB-1 (32, 33) has been previously reported to be infected with both MDV-1 strain BC-1 and MDV-2 strain HPRS-24 (34, 35). MDV-encoded glycoprotein B (gB), a highly conserved structural protein among alphaherpesviruses, is essential for virus entry (36), and we have previously shown that CRISPR/Cas9-based editing of gB prevented HVT infectivity (32). In this report, we have applied the double-sgRNA mediated targeted gene editing to delete MDV-2 gB gene from MSB-1 cells. Our studies show that deletion of MDV-2 gB in MSB-1 cells resulted in complete abolition of MDV-2 replication in co-cultivated chicken embryo fibroblast (CEF), while MDV-1 replication remained unaffected. Continued proliferation of the MDV-2-gB knockout cell lines confirmed that the MDV-2 gB gene is not essential for maintenance of the transformed state of MSB-1 cell line.

## RESULTS

### MDV-2 gB deletion in MSB-1.

**FIG 1:**
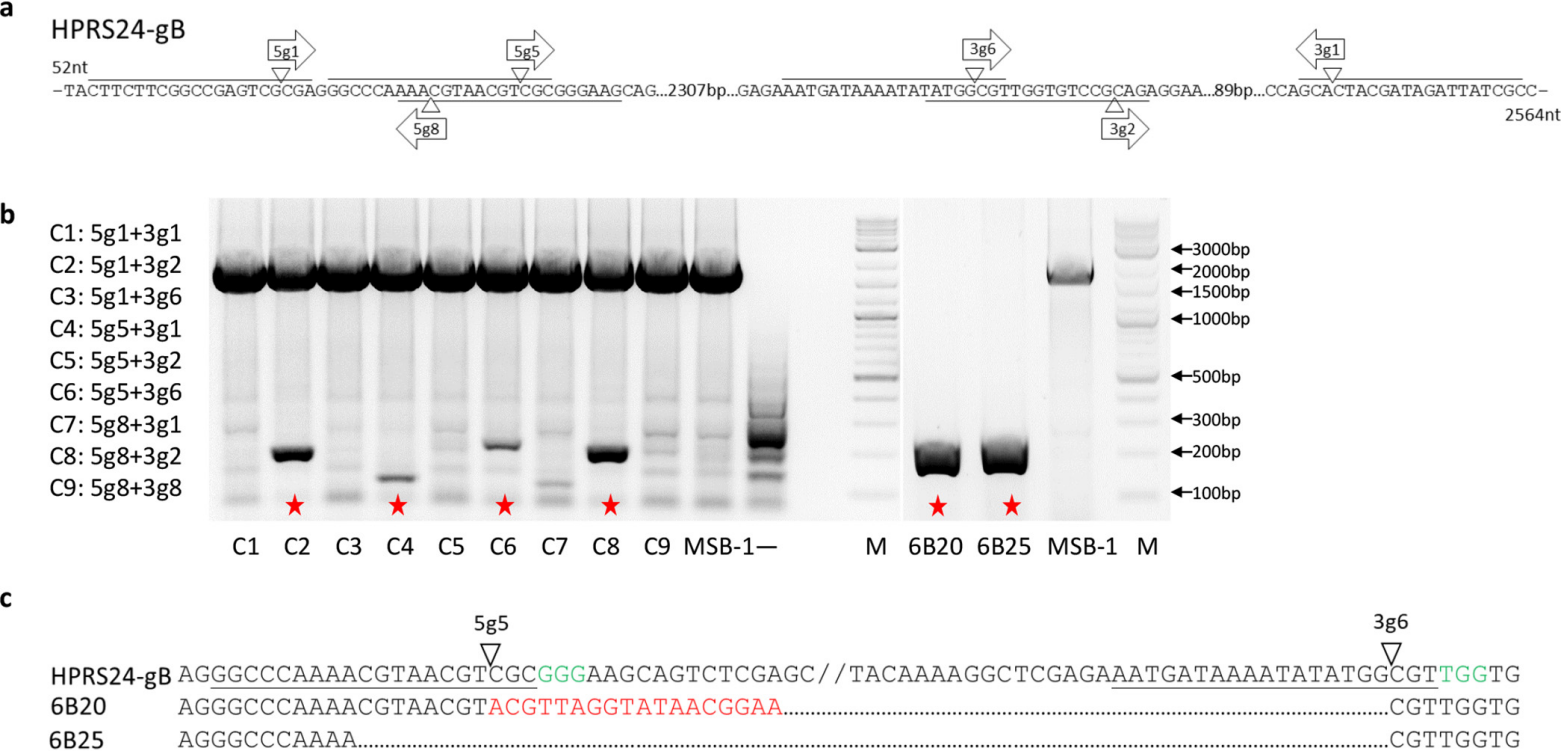
Deletion of MDV-2-gB by CRISPR/Cas9 editing in MSB-1 cells. (a) Three gRNAs from each end of MDV-2-gB were designed. The direction of each gRNA is indicated by arrows. Target sequence is underlined/overlined, and the cleavage site is indicated by pointers. Nucleic acid numbers for the gB gene are also indicated. (b) PCR amplification of the edited region with primers gB-N-F and gB-C-R on the cell lysates of transfected MSB-1 cells using different pairs of gRNAs (C1 to C9) at 24 h posttransfection (left) and on isolated single-cell clones 6B20 and 6B25 (right). Red asterisk indicates the transfected cells (left) or single cell clones (right) containing the edited band. The unedited MSB-1 was also included as control. (c) The nucleic acid sequences of the truncated/edited PCR product showing the successful deletion of MDV-2 gB. Target sequence is underlined, PAM sequence is in green and the cleavage site is indicated by a pointer. The inserted sequence in clone 6B20 induced by NHEJ repair pathway is in red.

Previously, we have reported on the efficient deletion of pp38 gene from MSB-1 using a dual gRNA construct expressing Cas9 nuclease and two gRNAs targeting both ends of pp38 gene in pX330-1 × 2 vector (20). Based on this success, we used the same approach for deleting MDV-2-gB from MSB-1. For this, we designed three gRNAs from each end of gB using the online gRNA designing tool and tested each pair of all possible combinations to maximize the chance of successful gB deletion (Fig. 1a). Nine pairs of different combinations using the three gRNAs from 5′ end (5g1, 5g5, and 5g8) and three gRNAs from 3′ end (3g1, 3g2, and 3g6) were cloned into pX330A-1 × 2 (Fig. 1b). For the targeted deletion of MDV-2 gB from MSB-1 cells, we transfected the dual gRNA construct into MSB-1, and the editing efficiency was assessed by PCR using specific primers located at the flanking region of Cas9-targeting sites. DNA from unedited cells generated a 2,582 bp PCR product (Fig. 1b). In contrast, DNA from Cas9/gRNA-transfected cells should generate the unedited 2,582 bp PCR product and/or a smaller edited band that corresponded to the deleted region between the two Cas9 cleavage sites amplified from edited cell population. The sizes of the edited smaller bands could vary between the different pairs of gRNAs. As shown in Fig. 1b, smaller bands were generated from four out of nine gRNA pair combinations, with 199 bp from C2 (5g1 + 3g2), 117 bp from C4 (5g5 + 3g1), 232 bp from C6 (5g5 + 3g6), and 212 bp from C8 (5g8 + 3g2), respectively. No smaller bands were observed from the remaining five pairs gRNA transfection, indicating that only four of these gRNA pairs were effective in MDV-2-gB gene deletion.

Single cell clones of transfected/edited MSB-1 cells were then sorted and analyzed by PCR to assess the editing pattern at single cell level. Sixty-three single cell clones from C2 transfection, 110 from C4 transfection, 63 from C6 transfection, and 69 from C8 transfection were analyzed. As expected, most of the clones showed only the unedited band (data not shown). Out of 305 single cell clones from four transfections, only two clones, 6B20 and 6B25, from C6 transfection showed complete editing with only smaller band (Fig. 1b). Sequence analysis of the edited band confirmed that it represented the potential end joining product of the DNA ends from cleavage at the predicated Cas9 target sites (Fig. 1c).

### Deletion of MDV-2-gB in MSB-1 inhibits MDV-2 replication in co-cultivated CEF.

**FIG 2:**
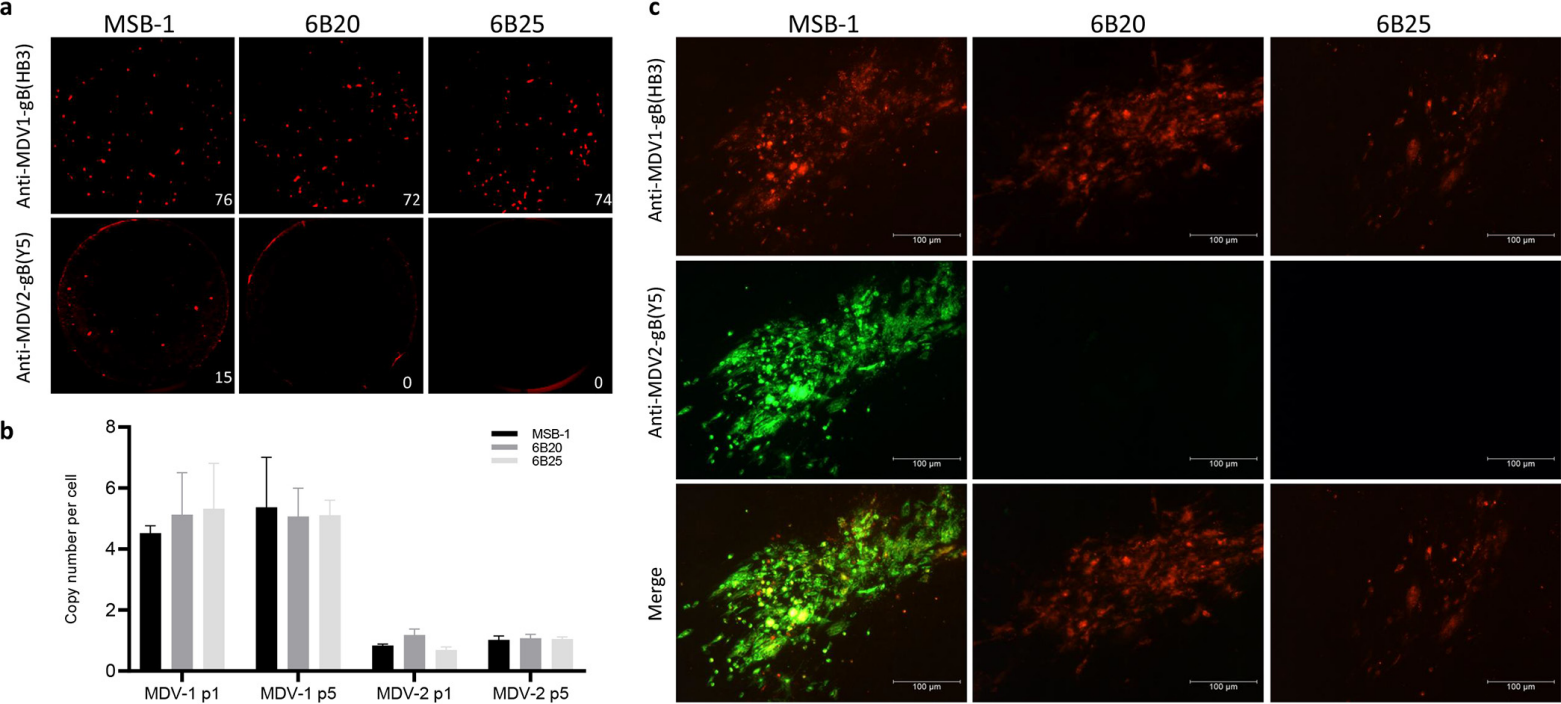
Inhibition of MDV-2 virus by successful deletion of MDV-2-gB in MSB-1 cells. (a) Detection of reactivating MDV-1 and MDV-2 by IFA using MDV-1 and MDV-2 gB-specific monoclonal antibodies HB3 and Y5, respectively, on plaques formed by co-cultivation of wild-type MSB-1, MDV-2-gB-deleted clones 6B20 or 6B25, and CEF. (b) MDV-1 and MDV-2 genome copies in MSB-1 and MSB-1-ΔMDV-2-gB cells at different passages measured by qPCR. (c) Detection of HB3-positive (red) MDV-1-gB-specific plaques in CEF co-cultivated with MSB-1 and MDV-2-gB-deleted cells, but Y5-positive (green) MDV-2-gB-specific plaques only in co-cultivated MSB-1 cells, confirmed the inhibition of MDV-2 in gB-deleted cells. The secondary antibodies are Alexa Fluor 488 goat anti-mouse IgG1 for MDV-2-gB expression and Alexa Fluor 568 goat anti-mouse IgG2b for MDV-1-gB expression. Pictures were taken with 100× magnification. The scale bar, 100 μm.

MSB-1 cells are latently infected with pathogenic BC-1 strain and non-pathogenic HPRS-24. Lytic replication and infectivity of these two viruses can be demonstrated from the low number of the virus plaques on primary CEF when co-cultivated with MSB-1 cells. We speculated that targeted deletion of the essential gB gene from the latent HPRS-24 genome in the MSB-1 should result in specific inhibition of MDV-2 plaques on co-cultivated CEF, with no effect on the MDV-1 plaques. To test this, we assessed the infectivity of the latent MDV-2 by co-cultivating the MDV-2-gB deleted MSB-1 clones 6B20 and 6B25 cells onto CEF. To increase the efficiency of latency to lytic switch, cells were treated with histone deacetylase (HDAC) inhibitor sodium butyrate (NaB) at 2.5 mM concentration for 48 h before co-cultivation of 10^6^ cells with CEF monolayer. Identity of the virus plaques formed on CEFs were confirmed by immunofluorescence assay (IFA) using MDV-1-gB-specific monoclonal antibody (MAb) HB3 and MDV-2-gB-specific MAb Y5. As demonstrated in Fig. 2a, the numbers of MDV-1-gB positive plaques formed on CEF were similar from both parental MSB-1 (*n* = 75 ± 5) and MDV-2-gB deleted MSB-1 clones 6B20 (*n* = 73 ± 8) and 6B25 (*n* = 71 ± 6). However, MDV-2 gB positive plaques were detected only in the parental MSB-1 (*n* = 16 ± 1), with no MDV-2 plaques detectable in the co-cultivated 6B20 and 6B25 cells, confirming the functional effects of MDV-2-gB deletion. Formation of fewer numbers of MDV-2 plaques in the co-cultivated MSB-1 cells compared with the numbers of MDV-1 plaques reflected the lower copy number of MDV-2 genome in relation to the MDV-1 genome copies in the MSB-1 cells, where approximately five MDV-1 copies and one MDV-2 copy are detected in MSB-1 cells (Fig. 2b). Importantly, the plaque numbers of the two viruses formed in cocultured CEF are proportional to the genome copy numbers in MSB-1. Moreover, detection of both MDV-1 and MDV-2 gB antigens in the virus plaques produced only with the co-cultivated parental MSB-1 cells (Fig. 2c), further confirmed the inhibition of MDV-2 growth as a result of the successful deletion of MDV-2-gB in 6B20 and 6B25 cells.

**FIG 3:**
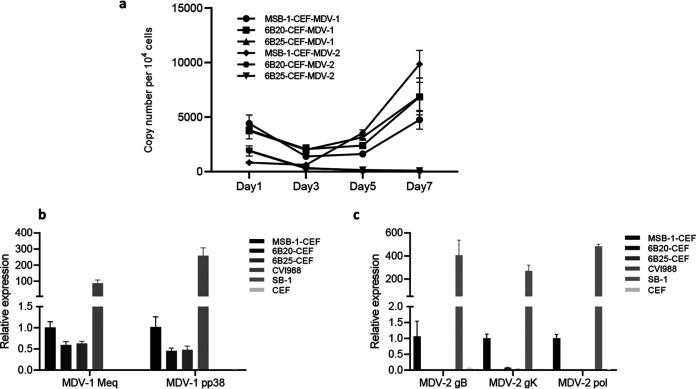
Deactivation of MDV-2 virus detected by qPCR and qRT-PCR. (a) MDV-1 and MDV-2 genome copies in CEF cocultured with MSB-1 and MSB-1-ΔMDV-2-gB clones measured by qPCR. Expression of MDV-1 genes Meq and pp38 (b) and MDV-2 genes gB, gK, and DNA-polymerase (c) measured by qRT-PCR (normalized to GAPDH) in RNA extracted from CEFs co-cultivated with MSB-1 and MSB-1-ΔMDV-2-gB clones 6B20 and 6B25. MDV-1 vaccine strain CVI988 and MDV-2 prototype vaccine strain SB-1 infected CEF were used as controls. Results represent the mean of triplicate assays with error bars showing the standard errors of the mean.

Having demonstrated that *in situ* deletion of the essential gB gene from the latent HPRS-24 genome results in targeted inhibition of MDV-2 replication in co-cultivated CEF (Fig. 2), we wanted to obtain further evidence of virus replication by measuring both viral genome replication and viral gene expression in CEF. For viral genome replication, we measured the dynamic changes of the viral genome copy numbers of both MDV-1 and MDV-2 in CEFs co-cultivated with MSB1 or MDV-2-gB deleted clones 6B20 and 6B25 at day 1, 3, 5, and 7 post-co-culturing by qPCR as described previously (37). As shown in Fig. 3a, the genome copy numbers were initially dropped for both MDV-1 and MDV-2 viruses in all cocultured cells, reflecting the gradual removal of the added cells attached to the CEF monolayer after continuous washing when CEFs were being harvested. The genome copy numbers of MDV-1 started increasing after 3 days in all three cocultured CEF samples, indicating the steady replication of the MDV-1 viruses after reactivation. In contrast, the viral genome copy number of MDV-2 only increased in MSB-1 but continued decreasing to undetectable level after 5 days in 6B20 and 6B25 where the MDV-2-gB has been deleted. These results clearly show that the reactivation of MDV-2 was inhibited when gB was deleted. For further confirmation of inhibition of MDV-2 virus replication, we analyzed the transcript levels of selected genes Meq and pp38 of MDV-1 and gB, gK and DNA polymerase of MDV-2 by quantitative RT-PCR in CEF co-cultivated with either parental MSB-1 or MDV-2-gB-deleted MSB-1 clones 6B20 and 6B25. As expected, expression of MDV-1 genes Meq and pp38 were readily detectable in MSB-1, 6B20 and 6B25 cocultured CEF (Fig. 3b), whereas the expression of MDV-2 genes was only detected in CEF co-cultivated with MSB-1, but not 6B20 and 6B25 (Fig. 3c). Absence of MDV-2-gB positive plaques, reduction in the copy numbers of the MDV-2 genomic DNA and lack of detectable MDV-2 gene expression by qRT-PCR in 6B20 and 6B25 cocultured CEF, further confirmed the functional consequences of the successful deletion of MDV-2-gB from MSB-1 cell line resulting in inhibition of the MDV-2 reactivation.

### MDV-2 gB is not essential in maintaining the transformed phenotype of MSB-1.

**FIG 4:**
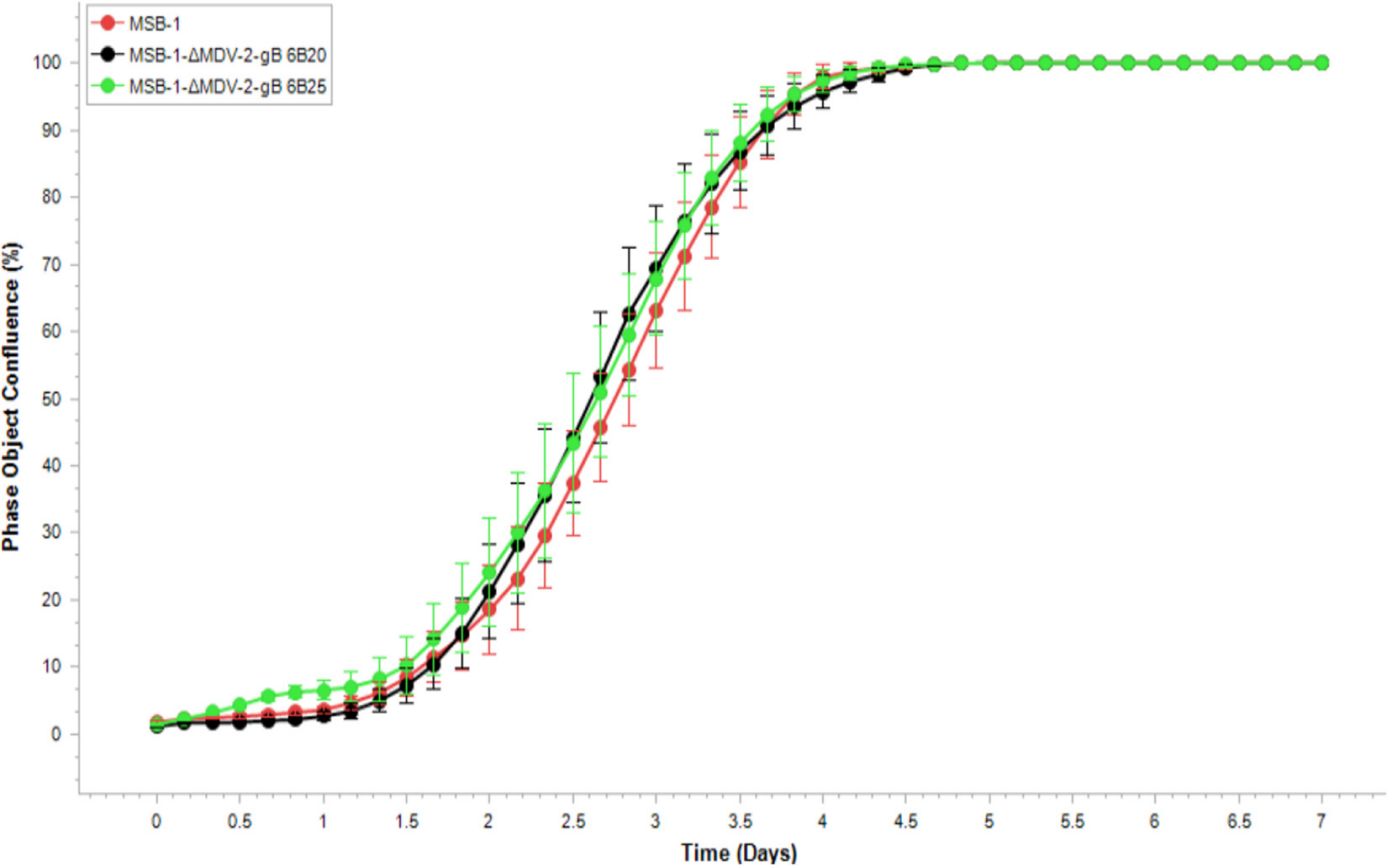
Proliferation of the MSB-1 and MSB-1-ΔMDV-2-gB clones monitored in real-time using IncuCyte S3 live imaging system. Cell phase object confluence of each cell population was determined every 4 h for 168 h from four separate regions per well and four wells per sample by IncuCyte S3 and compared with MSB-1 control. Growth curves are shown as mean ± standard error (SE) representative of three independent experiments.

As an essential gene in all herpesviruses, gB is required for infectivity and functions in penetration of cells by promoting fusion of the virion and plasma membranes. In majority of MSB-1 cells, where both MDV-1 and MDV-2 are thought to be latent state, gB is not expressed. Based on this, we predicted that targeted deletion of MDV-2 gB will have no effect on the growth of MSB-1 cells. To test this, we carried out kinetic monitoring of proliferation of the wild type MSB-1 and the MDV-2-gB deleted clones 6B20 and 6B25 using IncuCyte S3 Live-Cell Imaging system. As shown in Fig. 4, the cell proliferation data in real time from the images collected at 4-h intervals showed that there was no significant difference between the MDV-2-gB deleted clones and parental MSB-1 cells. This result has confirmed the hypothesis that MDV-2-gB was not essential for the continued proliferation of the transformed cells.

### Effect of MDV-2-gB deletion on expression of selected viral genes and miRNAs.

**FIG 5:**
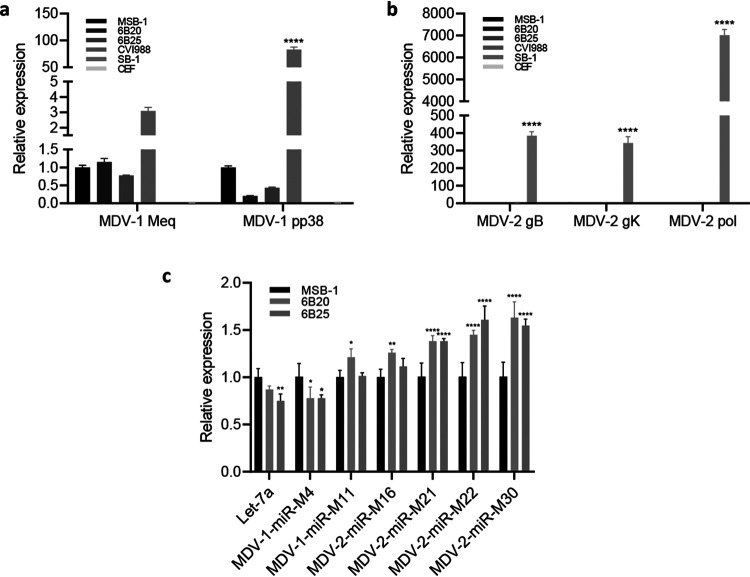
MDV miRNAs and viral gene expression in MSB-1 and MSB-1-ΔMDV-2-gB clones. Expression of MDV-1 genes Meq and pp38 (a) and MDV-2 genes gB, gK, and DNA polymerase (b) measured by qRT-PCR (normalized to GAPDH) in RNA extracted from MSB-1 and MSB-1-ΔMDV-2-gB clones. Results represent the mean of triplicate assays with error bars showing the standard errors of the mean. (c) Expression of MDV-1 miRNAs miR-M4, 11 and MDV-2 miRNAs miR-M16, 21, 22, 30, and host miRNA let-7a measured by qRT-PCR (normalized to U6 snRNA). Results represent the mean of triplicate assays with error bars showing the standard errors of the mean. Data was analyzed by two-way ANOVA (analysis of variance) with Tukey’s multiple comparisons using GraphPad Prism version 7.01. *, *P* < 0.05; **, *P* < 0.01; ***, *P* < 0.001; ****, *P* < 0.0001.

Having demonstrated that *in situ* deletion of MDV-2-gB in the MDV-transformed LCL MSB-1 resulted in the targeted inhibition of MDV-2 reactivation on co-cultivated CEF, we wanted to examine whether gB deletion had any effect on the expression of other MDV-encoded genes. For this, we compared the relative transcript levels of selected MDV-2 genes gB, gK and DNA polymerase in the parental MSB-1 and MDV-2-gB deleted clones 6B20 and 6B25. Additionally, we also examined the expression of MDV-1 gene Meq, one of the few expressed genes in MDV transformed cell line, and pp38 that are only expressed in minor population of spontaneously reactivated cells. CEF infected with MDV-2 vaccine strain SB-1 and MDV-1 vaccine strain CVI988 were used as the controls. Host gene GAPDH was included for normalization. As demonstrated in Fig. 5a, Meq and pp38 are still expressed although the level of pp38 expression decreased after deletion of MDV-2 gB. The absence of MDV-2 gB, gK and DNA polymerase genes before and after MDV-2-gB deletion demonstrated the latent nature of these genes that was unaffected by the deletion (Fig. 5b). We also examined the expression of host miRNA let-7a; MDV-1-encoded miRNAs MDV1-miR-M4 and miR-M11; MDV-2-encoded miRNAs MDV2-miR-M16; miR-M21; miR-M22; and miR-M30 on RNA extracted from parental and MDV-2-gB deleted MSB-1 cells. The expression of these miRNAs was normalized to the host U6 expression. As shown in Fig. 5c, the selected host and viral miRNAs are expressed before and after deletion of MDV-2-gB in MSB-1 although the expression level of MDV-2 miRNAs has increased. In addition, both MDV-1 and MDV-2 genome in the edited cells are stable as demonstrated by the same genome copy numbers of both MDV-1 and MDV-2 following continuous passages (Fig. 2b).

## DISCUSSION

CRISPR/Cas9 is revolutionizing genome editing approaches for a wide array of applications and across multiple disciplines due to its high efficiency, specificity, versatility, flexibility, simplicity, and low cost. Engineering viral genomes is becoming a key tool for studies of the structure and function of viral protein-coding genes and non-coding RNAs, virus-host interactions, development of recombinant vaccines, and gene therapy applications. MDV-transformed LCLs derived from MD lymphomas are valuable tools to study many aspects of virus-host interactions including transformation, latency, and reactivation. Compared with other MD LCLs, MSB-1 is distinct that it is infected not only by the pathogenic MDV-1 strain BC-1, but also by the non-pathogenic MDV-2 strain HPRS-24 (34). Although source of HPRS-24 infection in this cell line has not been identified, the bird from which MD lymphoma was induced may have already been infected with MDV-2 strains, as has been observed in many other clinical samples we have examined (unpublished data). As a non-pathogenic strain, it is unlikely that the MDV-2 strain had a direct role in the induction of lymphoma. However, the effects of the co-infected MDV-2 strain on the MSB-1 milieu, particularly on virus-host interactions in relation to the virus latency and reactivation, remain unclear. Targeted deletion of essential gene such as gB from the MDV-2 genome within the MSB-1 cells, could potentially be an approach to explore direct effects of MDV-2 in this cell line. We have recently described the application of CRISPR/Cas9 editing approach for *in situ* editing of the MDV-1 gene pp38 and miRNA MDV1-miR-M4 in LCL (20, 21). Here we use the same approach for targeted deletion of MDV-2 gB from MSB-1 cells to demonstrate the total inhibition of MDV-2 virus replication in co-cultivated CEF.

Several online tools and software for designing the gRNAs and providing a predictive value that ranks the gRNAs based on on-target and off-target activities are available. However, not all of gRNAs work well even with high-ranking scores (38, 39). Because of this, more gRNAs are often being tested to achieve the desired results. Gene disruption/knockout could be achieved by single gRNA, dual gRNAs, or multiple gRNAs. The number of gRNAs required for efficient gene disruption solely depends on the efficiency of gRNA being used. For example, a single gRNA was sufficient to disrupt GFP expression (40), and no escape mutant emerged when the GFP labeled HVT virus was targeted by this gRNA (41). A recent study has shown that the replication of very virulent MDV strain RB-1B was impaired by up to 50% using single gRNAs targeting the essential genes and was completely abrogated using multiple gRNAs (42). We have been using two gRNAs located either end of the gene successfully for knockout of MDV genes (20, 22) and miRNAs (21, 43). Our current study has shown that four out of nine gRNA pairs from different combinations of three high scoring gRNAs at either end of MDV-2 gB designed by Zhang Lab CRISPR design algorithm (http://crispr.mit.edu) generated small bands, demonstrating successful cleavage of both targeting sites (Fig. 1). Despite this relatively high editing efficiency, only two single cell clones were isolated from one of the four edited populations. The low recovery rate of the edited clones agrees with our previous observations (21, 43).

Demonstration of MDV-1 (HB3^+^)- and MDV-2 (Y5^+^)-specific plaques respectively on CEF co-cultivated with MSB-1 (Fig. 2) has confirmed the previous reports on the dual infection in this cell line (34, 35). Absence of MDV-2 replication indicated by the lack of MDV-2 gB positive plaques detected by IFA (Fig. 2), the MDV-2 genomic DNA detected by qPCR, as well as the MDV-2 gB, gK and DNA polymerase-specific transcript detected by qRT-PCR (Fig. 3) when MDV-2 gB deleted MSB-1 clones cocultured with CEF have further confirmed that the reactivation of MDV-2 has been abolished completely by the MDV-2 gB deletion in MSB-1. As gB is a latent gene in MDV transformed cells, it is not surprising that the proliferation of MSB-1 with MDV-2 gB deletion was not affected (Fig. 4). Our study also evaluated the effect of MDV-2 gB deletion on the expression of selected viral genes and miRNAs. MDV-1-encoded pp38 is a lytic gene expressed only in cells with reactivating virus, and the decreased level of pp38 expression reflects that lower percentage of such cells. MDV-2 in the MSB-1 cells is also considered to be in a latent state as evidenced by the absence of expression of gB, gK and DNA polymerase genes (Fig. 5b). On the other hand, we did see the expression of MDV-1 and MDV-2 miRNAs, as demonstrated previously (35).

In this study, we have demonstrated the successful use of CRISPR/Cas9 editing for selective inhibition of a fully infectious herpesvirus from a virus-transformed cancer cell line. Application of gene editing has been explored as an antiviral tool to cure infected cells of different viruses (44). Studies on inhibition of MDV replication *in vitro* and *in vivo* using CRISPR/Cas9 editing have become attractive in developing new intervention strategies in MD control. Hagag et al. has shown that combining gRNAs targeting two or more essential genes of MDV-1 completely abrogated virus replication and no escape mutants were observed upon serial passaging (42). A more recent study has demonstrated significant *in vivo* inhibition of MDV by expressing Cas9 and gRNA against ICP4 in transgenic chickens (45). In the current study, it is demonstrated that two gRNAs are sufficient to abolish the reactivation of MDV-2 virus. The approach described here can be used not only for identification of MDV genes involved in reactivation, but also for identification of essential genes for virus replication. The identified viral genes critical for inhibition of viruses can be used as targets for development of *de novo* disease resistance in chickens to avian viral pathogens, including other tumorigenic viruses. The strategy should also facilitate future analysis of the role of individual MDV genes and host genes in latency, transformation, reactivation, and host-virus interactions.

## MATERIALS AND METHODS

### Cell culture and viruses.

CEF used in this study were prepared from 10-day old Valo SPF embryos (ValoBioMedia GmbH, Osterholz-Scharmbeck, Germany). Cells were cultured in M199 medium (Life Technologies, Paisley, UK), supplemented with 5% fetal bovine serum (FBS, Sigma, Dorset, UK), 100 units/mL of penicillin and streptomycin (Life Technology), 0.25 μg/mL Fungizone (Sigma), and 10% TPB (tryptose phosphate broth, Sigma). A commercial SB-1 vaccine strain (SB-Vac) of MDV-2 used in this study is from Intervet UK Ltd. (Milton Keynes, UK). The MDV-transformed MSB-1 LCL from a spleen lymphoma induced by the BC-1strain of MDV (33) was grown at 38.5°C in 5% CO_2_ in RPMI 1640 medium (Thermo Fisher Scientific) containing 10% fetal bovine serum, 10% TPB, 1% sodium pyruvate solution (Sigma), and 100 units/mL of penicillin and streptomycin.

### Construction of gRNA constructs.

The Zhang Lab CRISPR design algorithm (http://crispr.mit.edu/) was used to design guide RNAs targeting both ends of MDV-2-gB gene of HPRS-24 strain. Three highest scoring gRNAs with a low probability of off target cleavage events from each end of gB sequence were chosen. Nine pairs of different combinations were cloned into CRISPR/Cas9 dual gRNA vector pX330A-1 × 2 as described previously (22). The oligonucleotides used are listed in Table 1.

**TABLE 1:** List of primer sequences

Primer	Sequence (5′–3′)
5g1-F	CACCGTTCTTCGGCCGAGTCGCGA
5g1-R	AAACTCGCGACTCGGCCGAAGAAC
5g5-F	CACCGGCCCAAAACGTAACGTCGC
5g5-R	AAACGCGACGTTACGTTTTGGGCC
5g8-F	CACCGTTCCCGCGACGTTACGTTT
5g8-R	AAACAAACGTAACGTCGCGGGAAC
3g1-F	CACCGCGATAATCTATCGTAGTGC
3g1-R	AAACGCACTACGATAGATTATCGC
3g2-F	CACCGATGGCGTTGGTGTCCGCAG
3g2-R	AAACCTGCGGACACCAACGCCATC
3g6-F	CACCGATGATAAAATATATGGCGT
3g6-R	AAACACGCCATATATTTTATCATC
gB-N-F gB-C-R	TCAGTGGGATCTGCGTTCCA TCCGAATCAGAGTACACAGGC
MDV-1-pp38-F	GATTCCACCTCCCCAGAATCC
MDV-1-pp38-R	CAGAGAATGCAACAATGCGT
MDV-1-Meq-F	GGTCTGGTGGTTTCCAGGTGA
MDV-1-Meq-R	GCATAGACGATGTGCTGCTGA
MDV-2-gB-F	AATTCAACACCCCCGAGTCC
MDV-2-gB-R	ACGCCATAAAACGGGGACAT
MDV-2-gK-F	GTTTGCAGAAGTTGCCCCAG
MDV-2-gK-R	TCGTTCATATCGCACGAGGG
MDV-2-pol-F	GCATGCGGGAAGAAAAGAG
MDV-2-pol-R	GAAAGGTTTTCCGCTCCCATA
GAPDH-F	GTCAACGGATTTGGCCGTAT
GAPDH-R	CCACTTGGACTTTGCCAGAGA
ovoF	CACTGCCACTGGGCTCTGT
ovoR	GCAATGGCAATAAACCTCCAA

### Generation and characterization of MSB-1-ΔMDV-2-gB cell line.

NEPA21 Electroporator (Sonidel Limited, Dublin, Ireland) was used for the transfection of MSB-1 cells. For the deletion of MDV-2-gB, 1 × 10^6^ of MSB-1 were resuspended in 96 μL Opti-MEM medium (Thermo Fisher Scientific) and mixed with 10 μg of Cas9/gRNA expression plasmid in 4 μL Opti-MEM to make a total volume of 100 μL and electroporated with optimized condition at voltage 175 V and a pulse width 1 ms of poring pulse for MSB-1. At 24 h postelectroporation, 1 × 10^5^ cells were harvested and analyzed by PCR using primers gB-N-F and gB-C-R followed by single cells sorting into 96 wells. After 7 days incubation, cells were collected and analyzed by PCR using 1 μL of the extracted DNA template with primers outside the targeting sites to identify the correct MDV-2-gB gene knocked-out clones.

### Single cell sorting.

For single cell cloning, cells were washed twice with PBS containing 5% FBS and centrifuged at 450 g for 5 min at room temperature. The cell pellets were resuspended in cold PBS/5% FBS and sorted into 96-well U-bottom plate (Corning) with growth medium by FACS using FACSAria II (BD bioscience).

### Growth of MSB-1-ΔMDV-2-gB cells.

The growth of MSB-1-ΔMDV-2-gB cells along with non-edited parental MSB-1 cells were monitored by IncuCyte S3 live cell imaging (Sartorius AG, Gottingen, Germany). Briefly, 8,000 cells were seeded in a 96-well plate and images were captured every 4 h for 168 h from four separate regions per well using a 10× objective. By recording the phase object confluence, the growth of MSB-1-ΔMDV-2-gB clones 6B20 and 6B25 were compared with parental MSB-1. IncuCyte data were analyzed by two-way ANOVA (analysis of variance) with Tukey’s multiple comparisons using GraphPad Prism version 7.01 (GraphPad Software, Inc., San Diego, CA, USA). The results were shown as mean ± standard error (SE) from four replicates each with four separate regions per well representative of three independent experiments.

### Reactivation of MDV from MSB-1-ΔMDV-2-gB cell line.

Following treatment with NaB at 2.5 mM concentration, 1 × 10^6^ of MSB-1 or mutant clones were co-cultivated with CEF monolayers in 6-well plates for 48 h (20). CEF monolayers were fixed with 4% paraformaldehyde and permeabilized with 0.1% Triton X-100 after 5 days incubation. The expression of MDV-2-gB was evaluated by IFA using fluorescence microscopy. The cells were stained with MDV-2-gB specific MAb Y5 (kindly provided by Dr. L.F. Lee, USDA-ADOL, MI) followed by secondary Alexa Fluor 488 goat anti-mouse IgG1 antibody for MDV-2-gB expression, and MDV-1-gB specific MAb HB3 (https://www.immunologicaltoolbox.co.uk/search?query=HB3) followed by secondary Alexa Fluor 568 goat anti-mouse IgG2b antibody for MDV-1-gB expression. Images were taken using a Leica DM IRB microscope (Leica Microsystems, Wetzlar, Hesse, Germany). Plaque number in 6-well plates was counted after staining with Y5 and HB3 followed by secondary antibody IRDye 680RD Goat anti-Mouse. The results were visualized using Odyssey Clx (Li-Cor).

### qRT-PCR analysis of miRNA and gene expression in MSB-1-ΔMDV-2-gB cells.

The expression level of miRNAs was analyzed using the TaqMan MicroRNA Assay System (Life Technologies) using 10 ng total RNA as a template for reverse transcription. Each reverse transcription reaction was tested by PCR in triplicate and performed twice independently. For relative quantification of host miRNA let-7a (Assay ID: 000377), MDV-encoded miRNAs MDV1-miRNA-M4 (Assay ID: 007333_mat), MDV1-miRNA-M11 (Assay ID: 005454_mat), MDV2-miRNA-M16 (Assay ID: 243682_mat), MDV2-miRNA-M21 (Assay ID: 006915_mat), MDV2-miRNA-M22 (Assay ID: 242599_mat), and MDV2-miRNA-M30 (Assay ID: 008351_mat) in MSB-1-ΔMDV-2-gB, all values were normalized to the expression of the endogenous U6 (Assay ID: 001973), and levels were calculated as fold-expression change relative to those from MSB-1. For relative quantification of viral genes in MSB-1-ΔMDV-2-gB and cocultured cells with CEF, all values were normalized to the expression of the endogenous GAPDH gene, and levels were calculated as fold-expression change relative to MSB-1 and MSB-1 cocultured with CEF, respectively.

### Determination of viral genome copy number.

Following treatment with NaB at 2.5 mM concentration for 48 h, 1 × 10^6^ of MSB-1 or mutant clones were co-cultivated with CEF monolayers in 6-well plates. After 24 h coculturing, the CEF cells were washed with PBS and cultured with fresh medium after removing the cocultured cells. At 1-, 3-, 5- and 7-days postinfection, infected cells were harvested after washing with PBS followed by DNA extraction using the DNeasy 96 blood and tissue kit (Qiagen, Manchester, UK) for real-time qPCR to determine MDV-1 and MDV-2 genome copy number, using the methods described previously (37). Real-time qPCR carried out to detect the MDV-1 Meq gene, MDV-2 polymerase, and the chicken Ovo transferrin gene enabled calculation of MDV-1 and MDV-2 genome copies per 10,000 cells using a dilution series of pCVI988 BAC DNA, pSB-1 BAC DNA, and p-GEM-T-ovo to produce a standard curve, respectively. The details of the primers which include MDV-1-Meq-F/MDV-1-Meq-R for Meq, MDV-2-pol-F/MDV-2-pol-R for MDV-2 polymerase, and ovoF/ovoR for ovotransferrin gene are listed in Table 1. PCR amplification was carried out in a 20-μL reaction volume with 10 μL of ABsolute Blue qPCR Low ROX Mix (Thermo Fisher Scientific), 0.5 μM forward and reverse primers, 0.2 μM probes, and 4 μL extracted DNA. The PCR conditions used were 95°C for 2 min, followed by 40 cycles at 95°C for 15 s and 60°C for 1 min. All qPCR tests were run in triplicate on the ABI 7500 Fast real-time PCR system (Thermo Fisher Scientific).

## References

[1] Davison AJ, Eberle R, Ehlers B, Hayward GS, McGeoch DJ, Minson AC, Pellett PE, Roizman B, Studdert MJ, Thiry E. 2009. The order herpesvirales. Arch Virol 154:171–177. 10.1007/s00705-008-0278-4. 19066710 PMC3552636

[2] Calnek BW. 1986. Marek’s disease–a model for herpesvirus oncology. Crit Rev Microbiol 12:293–320. 10.3109/10408418509104432. 3007026

[3] Osterrieder N, Kamil JP, Schumacher D, Tischer BK, Trapp S. 2006. Marek’s disease virus: from miasma to model. Nat Rev Microbiol 4:283–294. 10.1038/nrmicro1382. 16541136

[4] Neerukonda SN, Tavlarides-Hontz P, McCarthy F, Pendarvis K, Parcells MS. 2019. Comparison of the transcriptomes and proteomes of serum exosomes from Marek’s disease virus-vaccinated and protected and lymphoma-bearing chickens. Genes (Basel) 10:116. 10.3390/genes10020116. PMC6410298 30764491

[5] Witter RL. 2001. Protective efficacy of Marek’s disease vaccines. Curr Top Microbiol Immunol 255:57–90. 11217428 10.1007/978-3-642-56863-3_3

[6] Calnek BW, Schat KA, Peckham MC, Fabricant J. 1983. Field trials with a bivalent vaccine (HVT and SB-1) against Marek’s disease. Avian Dis 27:844–849. 10.2307/1590330. 6314982

[7] Suenaga T, Kohyama M, Hirayasu K, Arase H. 2014. Engineering large viral DNA genomes using the CRISPR-Cas9 system. Microbiol Immunol 58:513–522. 10.1111/1348-0421.12180. 25040500 PMC7168497

[8] Bi Y, Sun L, Gao D, Ding C, Li Z, Li Y, Cun W, Li Q. 2014. High-efficiency targeted editing of large viral genomes by RNA-guided nucleases. PLoS Pathog 10:e1004090. 10.1371/journal.ppat.1004090. 24788700 PMC4006927

[9] van der Oost J, Jore MM, Westra ER, Lundgren M, Brouns SJJ. 2009. CRISPR-based adaptive and heritable immunity in prokaryotes. Trends Biochem Sci 34:401–407. 10.1016/j.tibs.2009.05.002. 19646880

[10] Xu AT, Qin C, Lang Y, Wang MY, Lin MY, Li C, Zhang R, Tang J. 2015. A simple and rapid approach to manipulate pseudorabies virus genome by CRISPR/Cas9 system. Biotechnol Lett 37:1265–1272. 10.1007/s10529-015-1796-2. 25724716

[11] Liang X, Sun LQ, Yu T, Pan YF, Wang DD, Hu XY, Fu ZF, He QG, Cao G. 2016. A CRISPR/Cas9 and Cre/Lox system-based express vaccine development strategy against re-emerging Pseudorabies virus. Sci Rep 6:19176. 10.1038/srep19176. 26777545 PMC4726036

[12] Peng ZY, Ouyang T, Pang DX, Ma T, Chen XR, Guo N, Chen FW, Yuan L, Ouyang HS, Ren LZ. 2016. Pseudorabies virus can escape from CRISPR-Cas9-mediated inhibition. Virus Res 223:197–205. 10.1016/j.virusres.2016.08.001. 27507009

[13] Tang YD, Liu JT, Wang TY, An TQ, Sun MX, Wang SJ, Fang QQ, Hou LL, Tian ZJ, Cai XH. 2016. Live attenuated pseudorabies virus developed using the CRISPR/Cas9 system. Virus Res 225:33–39. 10.1016/j.virusres.2016.09.004. 27619840

[14] Yuan M, Zhang WS, Wang J, Al Yaghchi C, Ahmed J, Chard L, Lemoine NR, Wang YH. 2015. Efficiently editing the vaccinia virus genome by using the CRISPR-Cas9 system. J Virol 89:5176–5179. 10.1128/JVI.00339-15. 25741005 PMC4403460

[15] Yuen KS, Chan CP, Wong NHM, Ho CH, Ho TH, Lei T, Deng W, Tsao SW, Chen HL, Kok KH, Jin DY. 2015. CRISPR/Cas9-mediated genome editing of Epstein Barr virus in human cells. J Gen Virol 96:626–636. 10.1099/jgv.0.000012. 25502645

[16] Bierle CJ, Anderholm KM, Ben Wang J, McVoy MA, Schleiss MR. 2016. Targeted mutagenesis of guinea pig cytomegalovirus using CRISPR/Cas9-mediated gene editing. J Virol 90:6989–6998. 10.1128/JVI.00139-16. 27226370 PMC4944286

[17] Zou Z, Huang K, Wei YM, Chen HC, Liu ZD, Jin ML. 2017. Construction of a highly efficient CRISPR/Cas9-mediated duck enteritis virus-based vaccine against H5N1 avian influenza virus and duck Tembusu virus infection. Sci Rep 7. 10.1038/s41598-017-01554-1. PMC5431151 28469192

[18] Tang N, Zhang Y, Pedrera M, Chang P, Baigent S, Moffat K, Shen Z, Nair V, Yao Y. 2018. A simple and rapid approach to develop recombinant avian herpesvirus vectored vaccines using CRISPR/Cas9 system. Vaccine 36:716–722. 10.1016/j.vaccine.2017.12.025. 29269155 PMC5783714

[19] Tang N, Zhang Y, Sadigh Y, Moffat K, Shen Z, Nair V, Yao Y. 2020. Generation of a triple insert live avian herpesvirus vectored vaccine using CRISPR/Cas9-based gene editing. Vaccines (Basel) 8:97. 10.3390/vaccines8010097. PMC7157232 32098149

[20] Zhang Y, Luo J, Tang N, Teng M, Reddy V, Moffat K, Shen Z, Nair V, Yao Y. 2019. Targeted editing of the pp38 gene in Marek’s disease virus-transformed cell lines using CRISPR/Cas9 System. Viruses 11:391. 10.3390/v11050391. PMC6563304 31027375

[21] Zhang Y, Tang N, Luo J, Teng M, Moffat K, Shen Z, Watson M, Nair V, Yao Y. 2019. Marek’s disease virus-encoded MicroRNA 155 ortholog critical for the induction of lymphomas is not essential for the proliferation of transformed cell lines. J Virol 93. 10.1128/JVI.00713-19. PMC6694823 31189706

[22] Zhang Y, Tang N, Sadigh Y, Baigent S, Shen Z, Nair V, Yao Y. 2018. Application of CRISPR/Cas9 gene editing system on MDV-1 genome for the study of gene function. Viruses 10:279. 10.3390/v10060279. PMC6024840 29794970

[23] van Diemen FR, Kruse EM, Hooykaas MJ, Bruggeling CE, Schurch AC, van Ham PM, Imhof SM, Nijhuis M, Wiertz EJ, Lebbink RJ. 2016. CRISPR/Cas9-mediated genome editing of herpesviruses limits productive and latent infections. PLoS Pathog 12:e1005701. 10.1371/journal.ppat.1005701. 27362483 PMC4928872

[24] Tso FY, West JT, Wood C. 2019. Reduction of Kaposi’s sarcoma-associated herpesvirus latency using CRISPR-Cas9 to edit the latency-associated nuclear antigen gene. J Virol 93. 10.1128/JVI.02183-18. PMC6430552 30651362

[25] Ebina H, Misawa N, Kanemura Y, Koyanagi Y. 2013. Harnessing the CRISPR/Cas9 system to disrupt latent HIV-1 provirus. Sci Rep 3:2510. 10.1038/srep02510. 23974631 PMC3752613

[26] Hu W, Kaminski R, Yang F, Zhang Y, Cosentino L, Li F, Luo B, Alvarez-Carbonell D, Garcia-Mesa Y, Karn J, Mo X, Khalili K. 2014. RNA-directed gene editing specifically eradicates latent and prevents new HIV-1 infection. Proc Natl Acad Sci USA 111:11461–11466. 10.1073/pnas.1405186111. 25049410 PMC4128125

[27] Liao HK, Gu Y, Diaz A, Marlett J, Takahashi Y, Li M, Suzuki K, Xu R, Hishida T, Chang CJ, Esteban CR, Young J, Izpisua Belmonte JC. 2015. Use of the CRISPR/Cas9 system as an intracellular defense against HIV-1 infection in human cells. Nat Commun 6:6413. 10.1038/ncomms7413. 25752527

[28] Wang G, Zhao N, Berkhout B, Das AT. 2018. CRISPR-Cas based antiviral strategies against HIV-1. Virus Res 244:321–332. 10.1016/j.virusres.2017.07.020. 28760348

[29] Mwangi WN, Smith LP, Baigent SJ, Beal RK, Nair V, Smith AL. 2011. Clonal structure of rapid-onset MDV-driven CD4+ lymphomas and responding CD8+ T cells. PLoS Pathog 7:e1001337. 10.1371/journal.ppat.1001337. 21573129 PMC3088711

[30] Brown AC, Nair V, Allday MJ. 2012. Epigenetic regulation of the latency-associated region of Marek’s disease virus in tumor-derived T-cell lines and primary lymphoma. J Virol 86:1683–1695. 10.1128/JVI.06113-11. 22090140 PMC3264385

[31] Mwangi WN, Vasoya D, Kgosana LB, Watson M, Nair V. 2017. Differentially expressed genes during spontaneous lytic switch of Marek’s disease virus in lymphoblastoid cell lines determined by global gene expression profiling. J Gen Virol 98:779–790. 10.1099/jgv.0.000744. 28475033 PMC5657026

[32] Akiyama Y, Kato S. 1974. Two cell lines from lymphomas of Marek’s disease. Biken J 17:105–116. 4616680

[33] Nazerian K, Witter RL. 1975. Properties of a chicken lymphoblastoid cell line from Marek’s disease tumor. J Natl Cancer Inst 54:453–458. 163334

[34] Hirai K, Yamada M, Arao Y, Kato S, Nii S. 1990. Replicating Marek’s disease virus (MDV) serotype 2 DNA with inserted MDV serotype 1 DNA sequences in a Marek’s disease lymphoblastoid cell line MSB1-41C. Arch Virol 114:153–165. 10.1007/BF01310745. 2173523

[35] Yao Y, Zhao Y, Xu H, Smith LP, Lawrie CH, Sewer A, Zavolan M, Nair V. 2007. Marek’s disease virus type 2 (MDV-2)-encoded microRNAs show no sequence conservation with those encoded by MDV-1. J Virol 81:7164–7170. 10.1128/JVI.00112-07. 17459919 PMC1933330

[36] Chi XJ, Lu YX, Zhao P, Li CG, Wang XJ, Wang M. 2013. Interaction domain of glycoproteins gB and gH of Marek’s disease virus and identification of an antiviral peptide with dual functions. PLoS One 8:e54761. 10.1371/journal.pone.0054761. 23405092 PMC3566115

[37] Baigent SJ, Petherbridge LJ, Howes K, Smith LP, Currie RJ, Nair VK. 2005. Absolute quantitation of Marek’s disease virus genome copy number in chicken feather and lymphocyte samples using real-time PCR. J Virol Methods 123:53–64. 10.1016/j.jviromet.2004.08.019. 15582699

[38] Liu X, Homma A, Sayadi J, Yang S, Ohashi J, Takumi T. 2016. Sequence features associated with the cleavage efficiency of CRISPR/Cas9 system. Sci Rep 6:19675. 10.1038/srep19675. 26813419 PMC4728555

[39] Pallares Masmitja M, Knodlseder N, Guell M. 2019. CRISPR-gRNA design. Methods Mol Biol 1961:3–11. 10.1007/978-1-4939-9170-9_1. 30912036

[40] Shalem O, Sanjana NE, Hartenian E, Shi X, Scott DA, Mikkelson T, Heckl D, Ebert BL, Root DE, Doench JG, Zhang F. 2014. Genome-scale CRISPR-Cas9 knockout screening in human cells. Science 343:84–87. 10.1126/science.1247005. 24336571 PMC4089965

[41] Yao Y, Bassett A, Nair V. 2016. Targeted editing of avian herpesvirus vaccine vector using CRISPR/Cas9 nucleases. J Vaccine & Technol 1:1–7.

[42] Hagag IT, Wight DJ, Bartsch D, Sid H, Jordan I, Bertzbach LD, Schusser B, Kaufer BB. 2020. Abrogation of Marek’s disease virus replication using CRISPR/Cas9. Sci Rep 10:10919. 10.1038/s41598-020-67951-1. 32616820 PMC7331644

[43] Luo J, Teng M, Zai X, Tang N, Zhang Y, Mandviwala A, Reddy V, Baigent S, Yao Y, Nair V. 2020. Efficient mutagenesis of Marek’s disease virus-encoded microRNAs using a CRISPR/Cas9-based gene editing system. Viruses 12:466. 10.3390/v12040466. PMC7232411 32325942

[44] Kennedy EM, Cullen BR. 2017. Gene editing: A new tool for viral disease. Annu Rev Med 68:401–411. 10.1146/annurev-med-051215-031129. 27576009

[45] Challagulla A, Jenkins KA, O’Neil TE, Shi S, Morris KR, Wise TG, Paradkar PN, Tizard ML, Doran TJ, Schat KA. 2021. In vivo inhibition of Marek’s disease virus in transgenic chickens expressing Cas9 and gRNA against ICP4. Microorganisms 9:164. 10.3390/microorganisms9010164. 33450980 PMC7828426

